# Anti-Obesity Effects of Formulated Biscuits Supplemented with Date’s Fiber; Agro-Waste Products Used as a Potent Functional Food

**DOI:** 10.3390/nu14245315

**Published:** 2022-12-14

**Authors:** Thamer Aljutaily, Alaa Elbeltagy, Asmahan A. Ali, Mohamed G. E. Gadallah, Nazeha A. Khalil

**Affiliations:** 1 Department of Food Science and Human Nutrition, College of Agriculture and Veterinary Medicine, Qassim University, Buraydah 51452, Saudi Arabia; 2 Department of Food Science and Technology, Faculty of Agriculture, Menoufia University, Shibin El Kom 6131567, Egypt; 3 Department of Food Science and Nutrition, College of Science, Taif University, P.O. Box 11099, Taif 21944, Saudi Arabia; 4 Food Science Department, Faculty of Agriculture, Ain-Shams University, Cairo 11566, Egypt; 5 Nutrition and Food Sciences Department, Faculty of Home Economics, Menoufia University, Shibin El Kom 6131567, Egypt

**Keywords:** date powder, by-products, body weight, lipid profile

## Abstract

Superabundant date fruit production in Al-Qassim in the Kingdom of Saudi Arabia (KSA), a plentiful region for producing date syrup resulting in massive amounts of date fiber (DF), causes environmental issues with what is considered dietary waste. However, no food producer or researcher has thought of the valorization of DF by extracting the crude polysaccharides that can be converted to nanoparticles (flours) to increase its functional group and enhance its functionality. Using the DF was the primary goal, with the new biscuits used within the current study investigated as a potent integrated approach for controlling obesity levels and its effects. Obesity is one of the most important human problems worldwide, connected to many metabolic diseases, e.g., diabetes mellitus and cardiovascular disease. Its prevalence has recently increased among Saudi children and adolescents. An investigation of the biological effects of the formulated products was carried out by feeding the formulated biscuits with different DF levels (5, 10 and 15%) to obese albino rats, in addition to positive and negative control groups, to evaluate the effect of a reduced calorie product on controlling their body weight and health stats (lipid profile, blood sugars, kidney and liver functions). The collected data showed that the most positive results were obtained from rats fed diets supplemented with 10% DF biscuits. All TCHO, TrGs, HDL, and HDL were decreased to the best levels in this group compared to the positive control group (148.23, 145.30, 37.50, and 81.67 vs. 238.37, 199.07, 62.57, and 135.99, respectively). To conclude, DF supplementation presented anti-obesity properties in animal models; however, more epidemiological trials are needed.

## 1. Introduction

The Al-Qassim area is a massive place for thousands of tons of date fruit production annually in the Kingdom of Saudi Arabia (KSA), containing about 8 million trees that, according to the 2030 governmental plan, will soon double. Date fruits have many healthy properties because they are a good source of nutrients such as phytochemicals and selenium with low fat and protein levels. They can decrease chsolesterol levels, risk of heart disease (cardiovascular diseases; CVD), and cancer, additionally controlling diabetic patients’ glycemic index (diabetes mellitus; DM) and oxidative stress levels, thus preventing diabetic complications, e.g., atherosclerosis and neurodegenerative diseases [1,2,3,4,5]. On the other hand, such fruits are responsible for large amounts of crude date fiber (DF), a dietary waste causing many ecological problems. A few researchers have studied the functional characteristics and the related beneficial properties of such fruits and their derived by-products, e.g., waste date pulp, low-grade rejected date fruits, and date seeds (both pits and stones) [6,7,8].

Additionally, DF could boost different immune responses and gut health states using either soluble or insoluble fermentation substrates previously known as prebiotics (non-digestible oligosaccharides) [9]. Indeed, some recent studies by [10,11] demonstrated such a correlation for energy homeostasis improvement between plant-based diets high in fibers and polyphenolics used as a therapeutic diet via mechanisms of colonic microbiota (prebiotics and probiotics interactions). Furthermore, the recent innovative study by our team [12] used different DF levels in new highly valued supplemented biscuits that could be used as a value-added food for their prebiotic activities, valorizing their economic value. Indeed, recently published data show lifestyle changes have increased obesity prevalence in children and adults, so obesity correlates well with increasing different diets such as the Western diet [10]. Therefore, exchanging life dietary habits and styles could be more helpful for modulating many health problems such as obesity, not only among Saudi Arabian communities but also for most obese subjects worldwide.

Many different drugs have been used over the last three decades to reduce obesity by decreasing dietary fat preference. Orlistat is an important drug used for fat absorption reduction to lose energy but it may produce equally adverse effects such as oily stools or increased flatus that are associated with low levels of dietary fat consumption in favor of less energy-dense foods [9,13,14,15]. Moreover, a positive correlation between dietary fiber consumption and the reduction of coronary heart disease and diabetes incidence has been found. Additionally, it reportedly has a protective factor against degenerative disorders, especially obesity, with its consequences. For controlling and modulating human body weight using obese models, a recent innovative functional food is an exciting research area in the processed food industry. According to the world market analysis for functional foods, the demand for functional food in 2020 reached about USD 188,56 billion and is expected to increase to USD 27,577 billion by 2025. So, increasing the consumption of DF could help modulate such conditions (reduce obesity rate with its nutritional value and prebiotic effects). Indeed, previous data has proven that dietary fiber supplementations at high levels provide different beneficial effects, especially metabolic homeostasis including both reduced body weight and adiposity [10,16].

Weight, cholesterol, blood sugar levels, and renal function were monitored in obese albino rats to determine the biological effects of specially made, low-calorie biscuits. We do have a real issue here. Thus, we must find the best solution(s). So far as we are aware, no study has been conducted using date fiber (DF) to create anti-obesity effects. To that end, the current research aimed to assess the efficacy of three different DF doses (15, 10, and 5%) in a unique biscuit formulation designed to control appetite and satiety.

## 2. Materials and Methods

### 2.1. Chemicals

All obtained chemicals we used were from Pharmaceutical Science Laboratory, National Research Centre, Giza, Egypt, while starch used to feed experimental animals was from Edwic, Egypt, and casein, cellulose, and minerals. However, the vitamins used were purchased from Roche Vitamins and Fine Chemicals (Hague Rd, IN, USA), while cholic acid was from Sigma Company (Ronkonkoma, NY, USA).

### 2.2. Methods

#### 2.2.1. Biscuit Samples Preparation

Sukkari date fruit powder was subjected to fiber extraction by syrup separation as described by [17] was used for biscuit sample preparation (supplemented with 5, 10 and 15% DF) and left at room temperature to cool according to our previous recent publication [18] before being fed to the experimental animal models.

#### 2.2.2. The Study Design

Forty-eight male albino rats were purchased from the Vaccine and Immunity Organization of the Egyptian Ministry of Health’s Helwan Farm in Cairo. Every animal was housed in a clean, comfortable facility with a temperature of 25 °C, a 12:12 light:dark cycle, free access to water, and a basal (stander) meal for an entire week before the start of the experiment (healthy and ethical conditions were applied). All rats consumed the basal animal diet (standard diet), prepared according to AIN-93 guidelines [19] for seven days as an adaptation period [20], as illustrated in the Supplementary Materials Table S1 that also has the compositions of mineral and vitamin mixtures in the same Table S1. The animals, with an average weight of 200 g + 0.5 g, were raised in the animal house, faculty of Home Economics, Menoufia University, Egypt, after being approved by the ethical committee. They were divided randomly into six groups of eight rats and were kept under healthy normal conditions. Negative control non-obese group (C − ve; G1) and five obese groups; obesity was induced by high-fat diet (HFD; [21]) consumption for 4 weeks prior to the experiment. The five obese groups were divided into an obese positive group (C + ve; G2) and G3–6. The 3rd group was obese on a regular baseline diet (NBD) supplemented with Orlistat as a medical treatment for body weight loss. In contrast, G4, G5, and G6 were supplemented with 10% biscuit samples containing DF at different levels (5, 10 and 15%, respectively) for 30 days (Table 1).

Finally, the body weight was measured before and after the experiment to calculate the animals’ weight gain or reduction. At the end of the experiment, all the rats were sacrificed, and blood serum with kidney and liver were collected for blood glucose, lipid profile (total cholesterol, triglycerides, etc.) with kidney and liver functions for histopathology analysis. All were evaluated as described within the following sections.

#### 2.2.3. Body Weight Gain Calculation (BWG)

Biological evaluations of the different diets by determination of body weight gain % (BWG) and daily dietary intake were carried out and were calculated as described by [22] using the following formulas: BWG = final weight − initial weight while the differences from the control obese group (G2) were calculated as:

(1) $$\%\;\mathrm{BW}\;\mathrm{difference}=\frac{\mathrm{final}\;\mathrm{weight}\;\mathrm{of}\;\mathrm{each}\;\mathrm{group}\;-\;\mathrm{final}\;\mathrm{weight}\;\mathrm{of}\;\mathrm{group}\;}{G2\;\mathrm{final}\;\mathrm{weight}\;}\;\times \;100$$

#### 2.2.4. Sample Preparation for Biochemical Analysis

All collected blood samples were centrifuged to obtain their serum and then frozen for further biological analysis.

#### 2.2.5. Blood Biochemical Analysis

Collected blood samples at the end of the experiment were used for serum collection to measure the levels of blood glucose [23], lipid profile as indicated by total serum cholesterol (TCHO), triglycerides (TrGs), high-density lipoprotein cholesterol (HDL) as described previously according to [24,25,26]. Low-density lipoprotein cholesterol (LDL) and very low-density lipoprotein cholesterol (v-LDL) levels were determined using the methods of [27] as follows: LDL = total cholesterol − (HDL + v-LDL) while v-LDL = TG/5. 

Again, liver function as indicated by alanine aminotransferase (ALT) and aspartate aminotransferase (AST) was measured as previously described by [28]. Furthermore, as known by [29], urea and creatinine were determined as indicators of kidney function.

#### 2.2.6. Histopathology Analysis

At the end of the current study, experimental samples for histopathological examination were collected from kidney tissues to measure the effects of the treatments. They all were collected and fixed in neutral buffered formalin (10%), then routinely processed and embedded in paraffin wax. Paraffin blocks were sectioned at 4–5 um thickness and stained with Hematoxylin and Eosin [30].

#### 2.2.7. Statistical Analysis

Statistical differences were conducted with the SAS program [31], and all collected data were presented as means ± standard errors. All the analysis was performed statistically using a one-way analysis of variance (ANOVA and its extensions) followed by Duncan’s multiple range test, being considered statistically significant at *p* ≤ 0.05 [32].

## 3. Results

### 3.1. Body Weight Levels

Six animal model groups (G1:G6) were used to study the effect of biscuits supplemented with different DF levels (Diet 1–3; 5, 10 and15 DF %, respectively) on body weight reduction using obese models and all collected data were significantly (*p* ≤ 0.05) examined and are presented in Table 2. It can be seen from Table 2 that all the animal models used were at similar body weight levels at the beginning of the experiment for all obese models with no significant differences (about 220 gm; G2:G6), while the non-obese rat group (G1; C − ve) had the lowest initial weight, significantly (136 gm; *p* ≤ 0.05). Additionally, it can be seen that the different dietary fiber supplementations between the obese animal models which had the best effect on body weight reduction significantly (*p* ≤ 0.05) between the groups G6, G4, and G5 fed diets 3, 1 and 2, respectively, were losses of −74.34 followed by −32.00 and then −13.34 gm. Furthermore, for rats fed diet 3 (G6), there was an immediate effect on body weight reduction compared to the control obese rats fed Orlistat supplementation (G3); −74.34 and −89.00 gm, respectively. To conclude, obese rats fed biscuits with different dietary fiber concentrations had significant body weight reductions.

### 3.2. Blood Glucose Levels

The data in Table 3 represent the effects of the different biscuits supplemented with date fiber (DF) in different concentrations (diets 1–3; 5, 10 and15 DF %, respectively) on the blood glucose levels between the animal models (mg/dL) that were significantly examined (*p* ≤ 0.05). Table 3 illustrates that all the initial serum levels were at the lowest significant rate with G1, the non-obese rat group (107 mg/dL; *p* ≤ 0.05). However, the levels increased in all rat groups after feeding the HFD and starting the experiment. It was about 244 mg/dL for all the obese groups, G2–6, before feeding the examined diets. Regarding the final glucose levels, it should be noted that of the three DF supplementation levels in the animal models, the best decreased levels in their blood glucose were achieved by feeding diet 3 (G6) from the start point, and that it was very similar to the diet 2 (G5) effect (−135 and −134 mg/dL dietary DF, fed at 15 and 10%, respectively). Again, such an effect was similar when comparing the DF supplementations with the control-positive group (G2; C + ve), as both rat groups (G5 and G6) fed 10 and 15% DF were at a percentage reduction of about −57% of their blood glucose levels in comparison to levels obtained from the obese rats on a regular diet (C + ve; G2). To sum up, no significant differences were observed between obese animal models fed biscuits with 10 and 15% DF supplementation and animal models which consumed Orlistat supplementation (G3; *p* ≤ 0.05).

### 3.3. Lipid Profile Levels

Blood samples collected from all the experimental animal models were used to measure the effects of the biscuits supplemented at different DF levels for diets 1–3 (5, 10 and 15 DF % additions, respectively) on their lipid profiles; total cholesterol (TCHO), triglycerides (TrGs), low-density lipoprotein cholesterol (LDL), high-density lipoprotein cholesterol (HDL) and very low-density lipoprotein cholesterol (vLDL) and all collected data were examined for significant difference (*p* ≤ 0.05) and are presented on the following table (Table 4).

At the end of the experiment, the total cholesterol levels (TCHO) collected in Table 4 showed that the obese rats in G2 that were used as the obese positive control group (C + ve) fed on the regular diet had the highest TCHO levels (238.37 mg/dL) significantly within all the groups (*p* ≤ 0.05). In contrast, the negatively obese group fed on Orlistat (G3) had the lowest TCHO levels (146.60 mg/dL; Table 3) with significant differences between all groups except for the group fed diet 2; G5. The animal models fed diet 2 (biscuits supplemented with 10 DF %, G5) showed about 148.23 mg/dL serum total cholesterol; they were very close to G3 fed on Orlistat. In comparison, G4 and G6 had similar TCHO levels with no significant difference (fed biscuits with 5 and 15% DF, respectively; almost 157 mg/dL).

Regarding serum triglyceride (TrG) levels, again, Table 4 proves that rat group 2 that were the obese positive control group (C + ve) fed a regular diet with no supplementations had the most enormous significant (*p* ≤ 0.05) TrG levels among all the groups (199.07 mg/dL; G2). Again, the rat groups were fed the different treatments; biscuits supplemented with different DF amounts had TrG levels very close to each other with no significant differences. However, all were significantly different from the normal levels that were nearly the same in G1 and G3 (with no differences between them; 124.17 and 123.90 mg/dL, respectively). Additionally, the group that consumed diet 2 on biscuits with 10% DF supplementation had the lowest TrG levels 145.30 mg/dL). That group was followed by rats fed on diet 3; biscuits with 15% DF but very small differences (about 1 mg/dL TrGs; not significant). Finally, the obese rat group with diet 1 addition had the highest TrG levels; 149.43 mg/dL.

The calculated v-LDL levels (TrGs/5) are also shown in the same table for those worried about this issue (Table 4). The data showed that the v-LDL levels of the obese on a regular diet were the highest (about 48 mg/dL) in rat group 2. Obese rats given Orlistat were found to have similar blood sugar levels to the lean control (G1) rats, at roughly 30 mg/dL, although with significant differences (*p* ≤ 0.05). However, the lowest levels were recorded in group G5 (on diet 2; biscuits enriched with 10 percent DF), and there were no discernible differences in the levels in rats given Orlistat supplementation (G3; reached about 29 mg/dL), and the control group (G5).

Table 4 also presents the collected LDL data that was nearly the same between the rats that consumed biscuits supplemented with different levels of DF (diet 1 and diet 2) and the control on Orlistat groups (about 81 mg/dL) with no significant differences (*p* ≤ 0.05). The rats in group 2 (obese control on a regular diet) had the most considerable LDL levels (nearly 136 mg/dL).

Finally, the measured lipid profiles were then used for HLDL calculation; the levels are presented in the same table (Table 4). This data revealed that group 2, the obese on a regular diet, had the highest HDL levels of all groups (63 mg/dL approximately), while the non-obese control (G1) and obese rats fed Orlistat (G3) were close to each other, at about 37 mg/dL. However, the HDL levels seen in group 4 and group 6 (on diets 1 and 3; biscuits supplemented with 5 and 15% DF) reached about 38 mg/dL with no significant differences (*p* ≤ 0.05). The treated group that was least influenced was that with rats fed on diet 1 (G4; about 47 mg/dL; Table 4).

### 3.4. Kidney and Liver Functions

Table 5 illustrates the kidney (creatinine and urea) and liver (ALT and AST) function as mg/dL obtained from the blood serum samples from animal models fed biscuits supplemented with different DF levels. Table 5 shows that both elements used for measuring the kidney functions were at their highest significant levels in obese rats fed regular diets (G2) compared with all the other groups (positive control group; *p* ≤ 0.05). This test presented 42.33 and 1.24 mg/dL levels for both urea and creatinine, respectively. However, both elements were at their lowest levels in non-obese rats on a regular diet (G1; 24 and 0.5 mg/dL, respectively). Regarding the treatments used, it can be seen from the same table (Table 5) that the urea levels decreased in all the obese groups after biscuit sample supplementation at all the DF levels with no significant differences (diet 1–3; 5, 10 and 15% DF). Groups 5 and 6 showed the lowest urea levels, nearly the same at about 32 mg/dL. Additionally, creatinine levels were reduced significantly after consuming DF-supplemented biscuits, with no significant differences between rats fed diets 2 and 3 (0.78 and 0.67 mg/dL, respectively; Table 5). However, both groups were at the significantly lowest levels, compared with all the other rats.

Table 5 also explains the measured liver functions; AST and ALT. Both presented primary levels in the obese control rats fed a regular diet (C + ve; G2) of 120.80 and 104.60 mg/dL, respectively, which were significantly different from the other rat groups (*p* ≤ 0.05). However, the non-obese group rats (C − ve; G1) showed the deepest levels of both AST and ALT, 42.41 and 66.31 mg/dL, respectively, followed by rats in the group fed on Orlistat (G3; 45.73 and 64.73 mg/dL for AST and ALT, respectively). Again, G5, representing the obese rat group on diet 2 (biscuit sample supplementations at 10% DF) with 52.30 and 72.65, respectively, of AST and ALT, showed that this was the most effective treatment in comparison to the other treated groups.

### 3.5. Histopathology Analysis

**Figure 1:**
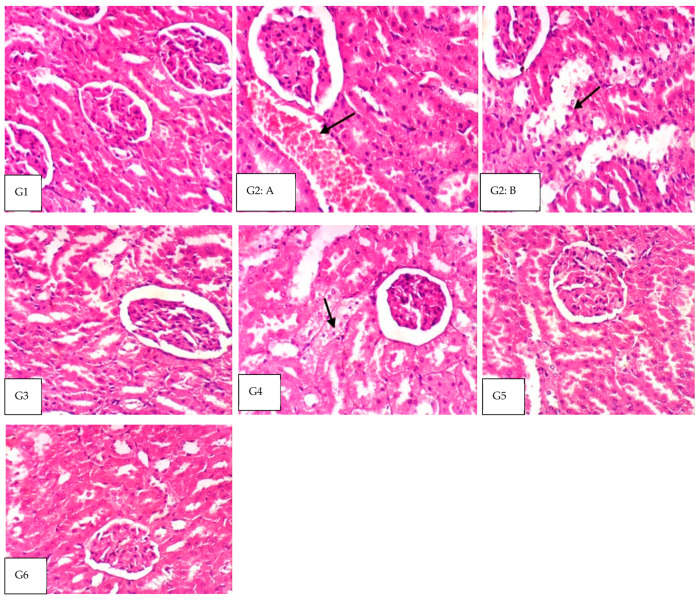
The histopathological kidney evaluation of rat groups; G1 shows the non-obese rats with normal histology of renal tissue (H&E X 400), G2K the obese on regular diet as G2: (A) presenting the congestion of renal blood vessels (H&E X 400) while G2K: (B) showing necrobiosis of the epithelial lining of some renal tubules (H&E X 400). Additionally, G3K of kidney of obese rats on Orlistat supplementation shows no histopathological alterations (H&E X 400). Furthermore, the kidney of obese rats fed on diet 1 is presented by G4K and shows vacuolation of epithelial lining in some renal tubules (H&E X 400). Finally, obese rats’ kidneys on diet 2 G5 and diet 3 showed no histopathological alterations (H&E X 400).

The current study measured the effects of the treatments used on the histopathology of kidney tissues. The data obtained from the rat groups revealed the following structure (Figure 1); the histopathology analysis of the kidneys obtained from different animal models fed biscuits supplemented with different DF levels. Microscopically, the kidneys of rats in G1 revealed the normal histology of renal tissue (Figure 1; G1), while the kidneys of rats from G2 showed vacuolation of epithelial lining renal tubules and congestion of renal blood vessels (Figure 1; G2:A) as well as necrobiosis of the epithelial lining of some renal tubules (Figure 1; G2:B). However, kidneys from G3 showed no histopathological alterations (Figure 1; G3). Vacuolation of the epithelial lining of some renal tubules was the only observed change in kidneys from G4 (Figure 1; G4). Meanwhile, examined sections from G5 and G6 revealed no histopathological alterations (Figure 1; G5 and G6).

## 4. Discussion

The control of human body weight is a fundamental issue, not only within Arabic countries but also worldwide, that has been controlled by following many complex and different strategies over years and years. Overweight and/or obesity problems depend on different reasons such as genetic, behavioral, environmental, and dietary factors [33]. The well-known diet for such a problem is the western diet, known to be an unhealthy diet with low dietary fiber levels (fruits, vegetables, beans, legumes, nuts, seeds, and whole grains) in contrast to high levels of sugars, meats, sweetened beverages in saturated fats and salt that, in combination, drive obesity and chronic diseases [34]. It is known that consuming a high fat and/or unbalanced diet and increasing and/or decreasing food intake levels will result in many different malnutrition diseases such as obesity, type 2 diabetes (T2D), cardiovascular diseases (CVD), and inflammatory bowel diseases (IBD), etc. Furthermore, different dietary fiber applications for improving and modulating human health significantly give protective effects against metabolic syndrome risk [35]. Indeed, a recent previous obesity study illustrated a significant decrease with increased dietary fiber intake. Many different weight-loss strategies have been implemented worldwide by using different dietary interactions. However, to our knowledge, no single study has been carried out using crude powdered date fiber (DF). Applying viscous fiber affected the human body weight and fat percentage in addition to the BMI levels [36]. Thus, high dietary fiber consumption has been associated well with low body weight. However, the amount of dietary fiber intake is not standardized worldwide with their portions and sizes. For instance, the British dietary guidelines for both genders aged 16–64 years old are 30 g/day, should be at least equal to five portions of fruits and vegetables, and whole grain cereals (pasta, bread, noodles), but this is not being achieved in the British population; they only consume about two portions [37].

Additionally, applicable dietary fibers have been measured for Finnish participants who presented high dietary cereal fiber intakes (67.8 g/day) in contrast to fibers obtained from fruit (3.3 g/day) and vegetables (6.5 g/day) that were much larger than those shown in other studies. Again, high consumption of dietary fiber presented generally at levels of more than 10 g/1000 kcal/day or about 20 g/day for human trials [16]. In contrast, a case-control dietary fiber study in Pamplona and Spain was split into different quintiles, starting from 19 g/day to 45 g/day [35].

Dates as a fruit are well known for most of the micronutrients; high levels of carbohydrates and minerals with significant amounts of calcium, iron, magnesium, phosphorus, potassium, and zinc, in addition to their substantial antioxidant and bioactive scavenging activities; in contrast to their low levels of fats and proteins that increase their nutritional values with healthy benefits [9,38,39]. It is an integral part of the Saudi diet with consumption in different kinds and processing in food industry areas resulting in a massive amount of date fiber becoming agro-waste products. Thus, the current innovative study interestingly aimed to study the anti-obesity effects of formulated biscuits supplemented with DF levels (5, 10, and 15%) for use as potential functional foods, produced from agro-waste products. The measured body weight, lipid profile, and blood sugars of obese albino rats were evaluated along with kidney and liver histopathology analysis.

Regarding the body weight of the animal models at the end of the current experiment, the final body weight changes to the initial body weight were shown to be the highest with the group fed on diet 3 and then diet 2 (biscuits with DF at 15 and 10%, respectively), close to the results from the rat group that consumed Orlistat that is well known for decreasing fat absorption resulting in energy loss in the feces. This could be attributed to the high DF concentration supplementation. Indeed weight loss and/or weight maintenance correlate well with different diets, such as low-fat and high-fiber diets, especially with vegetables and fruits containing high dietary fiber levels with low energy levels [9,40]. Different dietary fibers play an essential role with many significant therapeutic advantages and functional properties for human health, such as cholesterol absorption (limiting access to the body), gut-health promotion, and antidiabetic and anti-obesity properties [41].

The final blood glucose levels after feeding diet 3 (G6; dietary DF at 15%) showed again the best effectiveness of all three DF supplementations between the animal models as the levels reduced from the start point with very similar effect with no significant differences to diet 2 (G5; biscuits with 10% DF). Again, consuming high levels of DF-supplemented biscuits might be due to the dietary fibers’ beneficial effects and low fat and calorie levels. Indeed, different collected data have proven previously that rich dietary fiber consumption has antidiabetic effects in controlling blood glucose levels. For instance, eating soybean protein-rich fiber improved the glycemic index among type 2 diabetes mellitus patients [42]. Additionally, another study used Arabic gum consumption in diabetic animal models and showed blood glucose improvements due to their fiber levels [20].

The collected data from the measured lipid profile (TCHO, TrGs, v-LDL, LDL and HDL) demonstrated that animal models fed on diet 2 (biscuits supplemented with 10 DF %, G5) showed serum total cholesterol very close to those in G3 fed on Orlistat followed by both diet 1 and diet 3, the 5 and 15% additions. Again, the rat group consuming diet 2 on biscuits with 10% DF supplementation had the lowest TrGs levels, with a minimal difference from those rats fed on biscuits with 15% DF. Regarding the v-LDL, the groups fed on diet 2, biscuits supplemented with 10% DF (G5), showed the lowest levels as nearly the same with no significant differences from the Orlistat supplementation groups (G3). Additionally, measured LDL levels decreased in the animal models fed biscuits supplemented with different DF levels (diet 1 and diet 2; 5 and 10% DF, respectively). In contrast, HDL calculated levels were revealed as biggest in group 4 and group 6 (on diets 1 and 3; biscuits supplemented with 5 and 15% DF). Thus, all the different DF supplementation levels proved to have beneficial effects with significant positive effects in high doses or concentrated levels in good correlation and agreement with many previous studies. For example, numerous dietary fiber supplements used for weight loss promote and decrease the CVD risks that increase due to fat deposition in blood vessels by their higher LDL levels with special reductions in serum glucose and lipids [20,42,43,44]. Thus, the dietary date fiber used in powder form in the current study is considered an essential parameter with different functional anti-obesity properties resulting in body weight-, glucose-, and cholesterol-lowering levels and could be helpful for industrial food and/or product development.

The levels obtained for both urea and creatinine were used for kidney function measuring renal injury; high concentrated glucose levels could cause kidney damage with good indicators of renal dysfunction [45]. Indeed, both showed low levels; decreased in all obese groups after biscuit sample supplementation at all DF levels with no significant differences, especially groups 5 and 6 (lowest urea and creatinine levels; diets 2 and 3). Thus, the supplemented DF showed beneficial effects on kidney function due to body weight and glucose level reductions. Such data correlated well with some data from diabetic rat models that consumed antidiabetic dietary factors [18,46]. Additionally, the measured AST and ALT for liver function in the obese rat group fed biscuit sample supplementations at 10% DF (G5) showed this to be the most effective treatment compared to the results from the other treated groups.

At the end of the current study, the effects of the treatments (biscuits supplemented with different DF levels) on kidney tissue histopathology were presented in (Figure 1). Kidney of rats at G1 (the non-obese) and G3 (fed Orlistat) microscopically revealed normal renal tissue. In contrast, the kidneys of G2 (obese rats fed a regular diet) showed vacuolation of the epithelial lining renal tubules, congestion of the renal blood vessels, as well as necrobiosis of the epithelial lining of some renal tubules. Furthermore, the vacuolation of the epithelial lining of some renal tubules was only changed in kidneys from G4, with no histopathological alterations in the examined sections from G5 and G6. Indeed, the 30% obese models in the U.S. were previously associated with chronic kidney disease (CKD), resulting in hyperfiltration, albuminuria, and impairment in glomerular filtration rate in addition to correlation with the body weight increase [47]. Moreover, recent studies with obese individuals diagnosed with kidney disease have proven that glomerulomegaly and mesangial expansion resulted from high blood flow levels with hyperfiltration. High body weight gain causes many harmful pathways, simultaneously damaging both the glomeruli and the tubules, leading to progressive kidney injury [48,49,50]. Again, reducing human body weight can improve kidney function in association with a fat mass reduction [51]. Such correlation was seen in the current study, especially after dietary fiber supplementation associated with all the other measured parameters (blood creatinine and urea) resulting from body weight reduction, especially with G5 and G6 rats fed on diets 2 and 3 that had 10 and 15% DF supplemented levels. Such effective anti-obesity treatments may be attributed to the fiber constituents within the consumed biscuits at both 10 and 15 DF %. Consuming such levels should be rich with insoluble fibers that range from 84–94% and are mainly composed of cellulose, hemicelluloses, and lignin. The ratio of such insoluble fibers to the soluble ones is about 1:2, giving the dates beneficial physiological effects in addition to their antioxidant capacity. For instance, fiber contributes to viscous gel formation, especially in the intestine, causing the slowing of absorption of nutrients such as glucose and cholesterol and suppressing hunger for a long time. Additionally, it has more critical functional properties that can cause body weight reduction, such as water-holding and swelling capacity [52].

## 5. Conclusions

In summary, biscuits with DF supplementation at different levels for controlling body weight have proven to have hypoglycemic effects and anti-obesity actions with no histopathological alterations by improving the lipid profile, kidney and liver functions within the obese animal models used in contrast to the positive and negative control animal groups. Such effects resulted from their dietary fiber properties, especially the high levels of both 10 and 15% DF supplementation consumed. Therefore, such levels are highly recommended and could help in reformulation in the food industry to maintain body weight. However, long-term epidemiological trials are needed.

## Figures and Tables

**Table 1:** The study design and diets used with biscuits supplemented with different DF levels between groups of rats.

Animal Groups	NBD for 7 Days	HFD for 4 Weeks	Different Diets
Non-obese rats (C − ve; G1)	Yes	No	NBD
Obese on a normal diet (C + ve; G2)	Yes	Yes	NBD
Obese rats on Orlistat; G3	Yes	Yes	NBD + Orlistat
Obese rats on diet 1; G4	Yes	Yes	NBD + 10% biscuit (5% DF)
Obese rats on diet 2; G5	Yes	Yes	NBD + 10% biscuit (10% DF)
Obese rats on diet 3; G6	Yes	Yes	NBD + 10% biscuit (15% DF)

All rats were kept in standard conditions during all the experiments with 8 rats in each group. G means the group numbers: NBD, regular baseline diet; HFD, high-fat diet, while DF is date fiber used at different levels.

**Table 2:** Effects of biscuits supplemented with different DF levels on body weight levels of obese rats.

Animal Groups	Initial BW (gm)	Final BW (gm)	Differences (Final-Initial) BW (gm)	*p*-Value
Non-obese rats (C − ve; G1)	136.33 ± 2.72 ^b^	134.67 ± 2.60 ^d^	−1.67	0.000
Obese on normal diet (C + ve; G2)	221.00 ± 1.00 ^a^	237.00 ± 2.52 ^a^	16.00	
Obese rats on Orlistat; G3	220.00 ± 1.53 ^a^	131.00 ± 0.58 ^d^	−89.00	
Obese rats on diet 1; G4	219.00 ± 0.57 ^a^	251.00 ± 1.02 ^b^	−32.00	
Obese rats on diet 2; G5	219.33 ± 1.20 ^a^	232.67 ± 0.88 ^d^	−13.34	
Obese rats on diet 3; G6	218.67 ± 0.67 ^a^	144.33 ± 2.85 ^c^	−74.34	

Values are mean ± SE; *n* = 8. This means that the same column bearing different superscript letters is significantly different (*p* ≤ 0.05). ^a,b,c,d^ means with the same superscripted letters within the same column are not significantly different (*p* ≤ 0.05).

**Table 3:** Effects of biscuits supplemented with different DF levels on blood glucose levels between obese rats.

Animal Groups	Initial Glucose Levels (mg/dL)	Final Glucose Levels (mg/dL)	Differences (Final- Initial; mg/dL)	% Relative Change (G2; C + ve)
Non-obese rats (C − ve; G1)	107.00 ± 1.03 ^d^	106.67 ± 0.89 ^c^	−0.33	−58.76
Obese normal diet (C + ve; G2)	255.33 ± 1.67 ^a^	258.67 ± 0.69 ^a^	3.33	0.00
Obese rats on Orlistat; G3	249.67 ± 4.71 ^ab^	104.67 ± 0.76 ^c^	−145.00	−59.54
Obese rats on diet 1; G4	240.67 ± 3.72 ^c^	137.33 ± 0.66 ^b^	−103.33	−46.91
Obese rats on diet 2; G5	244.33 ± 1.75 ^bc^	110.33 ± 2.40 ^c^	−134.00	−57.35
Obese rats on diet 3; G6	245.67 ± 3.21 ^bc^	109.00 ± 0.37 ^c^	−135.33	−57.86

Values are mean ± SE; *n* = 8. ^a,b,c,d^ means with the same superscripted letters within the same column are not significantly different (*p* ≤ 0.05).

**Table 4:** Effects of biscuits supplemented at different DF levels on lipid profiles of obese rats.

Animal Groups	Lipid Profile Levels (mg/dL)				
	TCHO	TrGs	v-LDL	LDL	HDL
Non-obese rats (C − ve; G1)	153.77 ± 1.81 ^b^	124.17 ± 2.01 ^c^	30.75 ± 0.36 ^b^	92.41 ± 4.29 ^b^	36.53 ± 3.03 ^c^
Obese normal diet (C + ve; G2)	238.37 ± 0.38 ^a^	199.07 ± 1.24 ^a^	47.67 ± 0.08 ^a^	135.99 ± 1.05 ^a^	62.57 ± 0.47 ^a^
Obese rats on Orlistat; G3	146.60 ± 0.78 ^c^	123.90 ± 0.80 ^c^	29.32 ± 0.16 ^c^	84.32 ± 0.17 ^c^	37.50 ± 0.87 ^c^
Obese rats on diet 1; G4	157.33 ± 1.45 ^b^	149.43 ± 0.29 ^b^	31.47 ± 0.29 ^b^	80.58 ± 1.39 ^c^	46.87 ± 1.07 ^b^
Obese rats on diet 2; G5	148.23 ± 0.81 ^c^	145.30 ± 2.49 ^b^	29.64 ± 0.16 ^c^	81.67 ± 0.46 ^c^	37.50 ± 0.62 ^c^
Obese rats on diet 3; G6	157.17 ± 1.63 ^b^	146.67 ± 1.34 ^b^	31.43 ± 0.33 ^b^	87.87 ± 3.72 ^bc^	39.97 ± 2.26 ^c^

Values are mean ± SE; *n* = 8. TCHO: total serum cholesterol; TrGs: triglycerides; v-LDL: very low-density lipoprotein cholesterol; LDL: low-density lipoprotein cholesterol; HDL: High-density lipoprotein cholesterol, ^a,b,c^ means with the same superscripted letters within the same column are not significantly different (*p* ≤ 0.05).

**Table 5:** Effects of biscuits supplemented with different DF levels on kidney and liver functions in obese rats.

Animal Groups	Kidney (mg/dL)		Liver Functions (mg/dL)	
	Urea	Creatinine	AST	ALT
Non-obese rats (C − ve; G1)	24.00 ± 2.08 ^c^	0.50 ± 0.01 ^d^	42.41 ± 1.62 ^d^	66.31 ± 0.41 ^e^
Obese normal diet (C + ve; G2)	42.33 ± 1.45 ^a^	1.24 ± 0.01 ^a^	120.80 ± 2.60 ^a^	104.60 ± 1.63 ^a^
Obese rats on Orlistat; G3	26.80 ± 0.70 ^c^	0.52 ± 0.02 ^d^	45.73 ± 1.42 ^d^	64.73 ± 2.92 ^de^
Obese rats on diet 1; G4	37.00 ± 1.53 ^b^	0.94 ± 0.03 ^b^	87.87 ± 1.37 ^b^	80.51 ± 3.25 ^b^
Obese rats on diet 2; G5	32.67 ± 1.20 ^b^	0.78 ± 0.02 ^c^	52.30 ± 0.61 ^c^	72.65 ± 2.07 ^d^
Obese rats on diet 3; G6	32.33 ± 1.86 ^b^	0.67 ± 0.09 ^c^	65.83 ± 2.02 ^c^	74.03 ± 1.38 ^c^

Values are mean ± SE; *n* = 8. ^a,b,c,d,e^ means with the same superscripted letters within the same column are not significantly different (*p* ≤ 0.05).

## Data Availability

All relevant data used to support the current research findings are included within the article. The raw data are also available at the Department of Food Science and Human Nutrition, College of Agriculture and Veterinary Medicine, Qassim University, KSA.

## Supplementary Materials

The following supporting information can be downloaded at: https://www.mdpi.com/article/10.3390/nu14245315/s1, Table S1: Composition of basal diet ingredients (g/100 g diet), vitamin and mineral mixtures (g/1000 g).
