# Supplementary material for: Anti-Obesity Effects of Formulated Biscuits Supplemented with Date’s Fiber; Agro-Waste Products Used as a Potent Functional Food
Source: Nutrients. 2022 Dec 14;14(24):5315. doi: 10.3390/nu14245315 (PMC9783991; doi:10.3390/nu14245315)
Supplement: Supplementary file 1 [file nutrients-14-05315-s001.zip › nutrients-1979913-supplementary.pdf]

Supplemental Material

Table S1. Composition of basal diet ingredients (g/100g diet), vitamin and mineral mixtures (g/1000g).

Ingredient	Quantity(g)	Vitamins	Quantity(g)	Minerals	Quantity(g)
*Casein	12	Vitamin A	2000UL	$CaCO_3$	300
Sunflower oil	10.0	Vitamin D	200 UL	KH_2PO_4	322
Mineral-mixture	4	Vitamin K	10 UL	$CaPO_42H_2O$	75
Vitamins-mixture	1	Inositol	10 UL	$MgSO_47\ H_2O$	102
Fibers (cellulose)	4	Niacin	4 mg	NaCl	167
Sugar (sucrose)	10	Ca-Pantothenate	4 mg	$FeC_6H_6O_7\text{-}6H_2\ O$	27.5
DL- methionin	0.3	Riboflavin (B2)	0.08 mg	KI	0.9
Choline chloride	0.2	Thiamine (B1)	0.5 mg	$MnSO_4\ 4H_2O$	5.0
Corn starch	58.50	Pyridoxine	0.5 mg	$ZnCl_2$	0.25
		Folic acid	0.2 mg	$CuSO_4\ 5H_2O$	0.40
**Total**	**100 (g)**	Biotin	0.04 mg	**Total**	**1000g**
*12g casein yielded 10.32 g protein		Cyano cobalamin (B12)	0.03 mg		
		Choline chloride	200 mg		
		P-amino benzoic acid	10 mg		
